# KSHV infection of endothelial cells manipulates CXCR7-mediated signaling: implications for Kaposi’s Sarcoma progression and intervention

**DOI:** 10.1186/1750-9378-7-S1-O6

**Published:** 2012-04-19

**Authors:** Jennifer Vomaske, Lisa Clepper, Janet Douglas, Liron Pantanowitz, Klaus Fruh, Ashlee V Moses

**Affiliations:** 1 Vaccine and Gene Therapy Institute, Oregon Health & Science University, Portland, OR, USA; 2 Department of Pathology, University of Pittsburgh School of Medicine, Pittsburgh, PA, USA

## 

CXCR7 was recently characterized as an alternative receptor for the chemokine CXCL12/SDF-1, previously thought to bind and signal exclusively through CXCR4. We recently identified CXCR7 as a key cellular factor in the endothelial cell (EC) dysfunction associated with KSHV infection. CXCL12 signaling is critically associated with tumor growth, angiogenesis and metastasis in several diverse tumors and is one of the most studied chemokine/chemokine receptor interactions in cancer systems. The tumorigenic activity of the CXCL12 signaling axis offers an attractive target for therapeutic intervention in multiple cancers including Kaposi’s Sarcoma (KS). However, most of the research to date was based on the assumption that CXCR4 was the sole CXCL12 receptor, and thus focused on the development of CXCR4-targeted treatments. CXCR4 participates in important homeostatic functions including hematopoiesis and mucosal immunity, while CXCR7 is rarely expressed in normal adult cells. As a result, CXCR7 may be a more specific chemotherapeutic target for tumor cells and tumor-associated vasculature with fewer adverse effects than treatments targeting CXCR4. CXCR7 is poorly studied throughout the cancer literature and although CXCR7 expression has been found in tumor-associated vasculature, no studies comprehensively examine the biology of CXCR7 in EC and its implications for tumor biology. We seek to define the role of CXCR7-mediated CXCL12 signaling in EC biology, and in the context of KSHV infection, in order to determine potential contributions of CXCR7 signaling to KSHV-mediated EC transformation and KS tumorigenesis. We demonstrate that CXCR7 is strongly expressed on LANA+ spindle cells in KS biopsy tissue at all stages of tumor progression. We further demonstrate that CXCR7 induction by KSHV *in vitro* is specific to lymphatic EC lineages and occurs coincident with the acquisition of spindle morphology. Detailed examination of CXCR7 functions in EC biology reveals multiple roles for CXCR7 that could impact KS tumorigenesis, including effects on cellular proliferation, junctional integrity, cell survival and metastatic capacity. Specifically, we determine that CXCR7 expression results in a loss of PECAM/CD31 expression, perturbing the formation and maintenance of EC monolayers. Moreover, CXCR7+ EC display significant SDF-1 dependent hypermotility, as measured via Electrical Cell-Substrate Impedence Sensing (ECIS). We also demonstrate that SDF-1 signaling through CXCR7 expression is enhanced in EC undergoing anchorage-deprivation, affecting EC cell survival and invasion into SDF-1 rich niches. Taken together, these results demonstrate that CXCR7 is a novel KSHV-induced oncogene with the capacity to influence multiple aspects of KS pathogenesis including tumor growth, seeding and metastasis.

